# Methodological Considerations in Cognitive Training Research

**DOI:** 10.3389/fpsyg.2016.01481

**Published:** 2016-09-30

**Authors:** Joachim T. Operskalski, Aron K. Barbey

**Affiliations:** ^1^Decision Neuroscience Laboratory, University of Illinois at Urbana–ChampaignUrbana, IL, USA; ^2^Beckman Institute for Advanced Science and Technology, University of Illinois at Urbana–ChampaignUrbana, IL, USA; ^3^Neuroscience Program, University of Illinois at Urbana–ChampaignUrbana, IL, USA; ^4^Department of Psychology, University of Illinois at Urbana–ChampaignUrbana, IL, USA; ^5^Department of Speech and Hearing Science, University of Illinois at Urbana–ChampaignUrbana, IL, USA; ^6^Department of Internal Medicine, University of Illinois at Urbana–ChampaignUrbana, IL, USA; ^7^Carl R. Woese Institute for Genomic Biology, University of Illinois at Urbana–ChampaignUrbana, IL, USA; ^8^Carle Neuroscience Institute, Carle Foundation HospitalUrbana, IL, USA

**Keywords:** cognitive training

## Introduction

The goals of cognitive training programs resonate with us. Many of us have seen loved ones succumb to the ravages of Alzheimer's Disease, and others of us simply take pleasure in feeling mentally agile or watching as our children's minds grow seemingly overnight. We live every day confronted by the challenges of navigating a complex world, and most of us would like to be better at it. In many ways, scientific evidence supports the intuition that cognitive training should work; the brain is shaped by its experiences throughout the lifespan (Kramer et al., 2004; Markham and Greenough, 2004), and simply believing that intelligence is a malleable set of abilities confers the benefits of motivation and learning strategies that are known to correspond to higher performance on psychometric tests (Blackwell et al., 2007). As an academic and clinical community, if we seek to help people reach their full cognitive potentials, what better time could there be to bolster their abilities than during adolescence, when neurons in the brain are growing to their peak levels of axonal myelination, and when the synaptic connections between them are rapidly being strengthened or pared down by experience? Furthermore, how can we leverage what we know about neuroscience and psychology to even the playing field between people at risk for the negative developmental impacts of poverty and disease, and the more fortunate?. These are among the questions that Jacqueline Gamino and her colleagues at the UT Dallas Center for BrainHealth have been exploring in their Strategic Memory Advanced Reasoning Training (SMART) program (Gamino et al., 2014). These questions are also among those that will advance the fields of education policy and aging research alike, but we recommend caution in interpreting the most recent findings to emerge from the cognitive training field.

## Strategic memory advanced reasoning training (SMART)

Strategic Memory Advanced Reasoning Training (SMART), as implemented in the 2014 study by Gamino et al., is a 1-month training program created with the goal of teaching “hierarchical cognitive strategies that support higher-order abstraction of meaning from incoming details and world knowledge” (Gamino et al., 2010). It focuses on verbal comprehension for written texts, with a focus on nuanced meaning rather than memorization of explicit details, with seven components of instruction: “ (1) deliberate inhibition of extraneous information; (2) chunking and organizing relevant information; (3) inference; (4) paraphrasing; (5) synthesis of important details; (6) interpretation of take home messages; and (7) abstraction of deeper meanings and synthesis of the processes in order to elicit top-down processing” (Gamino et al., 2014).

One of the ideas motivating SMART is that to be useful, cognitive training should not involve practicing a single skill in a narrowly-defined context with limited applicability to daily life. This reflects one of the key criticisms of early cognitive training games, which was the notion of limited “transfer,” or whether gains made while practicing a video game generalized to real-life performance (far transfer) or at least to similar measures of the same ability (near transfer), rather than being accounted for solely by procedural memory for the task at hand. The SMART program addresses this concern by using a broad training platform with similarity to real-life academic demands as an alternative strategy to methods that involve practicing a single skill like memory span or selective attention. Gamino et al. also used outcome measures that were designed to measure the skills necessary for academic success beyond rote memorization. Instead of exhausting the search for “far transfer” between psychometric task-based training materials and the “tasks” encountered in real life, they created a training protocol closer to the demands of school performance. The advantage of this approach is that even if a program's benefits only go as far as “near transfer” or can even be attributed entirely to procedural memory mechanisms in the brain, they could be worthwhile if they help people meet their goals beyond the training program.

## Limitations in study design and interpretation

With the promise of reasoning training in mind, the next step is to evaluate the state of the current evidence. The most prominent finding in the recent study by Gamino et al. was that within the group of students who received the cognitive training program, they all showed similar improvements in verbal comprehension and abstract thought regardless of their socioeconomic status (SES); the students from families that were financially struggling improved over the course of the intervention. While these preliminary results should be followed up and studied further, the following key details need to be highlighted and addressed in future research if we aim to translate “hope for students living in poverty” into tangible benefits.

1. The analysis showing equal gains for students living above and below the poverty line used a statistical model adjusting for baseline performance levels in the outcome of interest and memory retrieval. The primary finding was the lack of an interaction between SES and time (pre-intervention vs. post-intervention), but it is important to note that students living in poverty scored lower than affluent students before the intervention, and there was no evidence presented for any closing of the achievement gap after the intervention. In other words, *students in poverty improved a presumably similar amount from a lower baseline*. Furthermore, Bayesian statistical analysis (or at least some consideration of standardized effect size) is more well-suited than the Neyman-Pearson hypothesis testing framework for interpreting what appears to be the lack of a difference between two groups (low-SES and high-SES students having similar within-group gains) as anything other than what we typically call “failure to reject the null hypothesis.”
2. The control group showed a non-significant improvement in their reasoning scores between the two assessments while the experimental group showed statistically significant improvements from baseline, but *the authors do not report whether there was a main effect of intervention group*. By analogy, if you (the reader) should score 101 on an IQ test while we score 99, then you are technically above the population average and we are below average, but this does not say anything about whether we are reliably different from one another without directly comparing our scores to each other instead of against an arbitrary significance threshold.
3. The control condition itself differed systematically from the experimental group in ways other than whether they received the intervention or not, making comparisons between the groups impossible. The interaction between SES and cognitive training (or a purported lack thereof) was thus demonstrated only within participants who received the cognitive training intervention. The intervention was administered to classes and schools where teachers and the administration volunteered to have class time devoted to the SMART program. *Baseline and follow-up testing was administered to control participants from schools (or specific classes) that were not willing to engage in the intervention*. If willingness to participate in what is described as a cutting-edge, neuroscience-based educational psychology intervention is any indicator of teaching style, then there could be systematic differences in the type of instruction students in the experimental and control conditions were already receiving. The control group also included a larger range of ages and a longer period of time between the two assessments. The authors explain that the schools participating in the experiment required that all students be given equal access to the intervention if it were beneficial. The well-intentioned school administrators may not understand the need to control intervention trials against a placebo or “treatment as usual”; if we already knew that the intervention were better than other educational methods in this particular context, then we would not be testing it. In vulnerable populations who deserve access to an intervention for which there is already building evidence, one ethical solution to this dilemma is to offer a staggered onset of treatment, in which the control group is assessed without intervention (or with instruction as usual) for the duration of the experiment before being offered the chance to receive the intervention immediately afterward.
4. Even if the contents of an intervention do not confer improvement in the brain's ability to process information or the adoption of adaptive strategies, *believing that one will improve can change motivation, attention, or other factors that will increase performance as well*, rendering the intervention itself unnecessary. This is reason to include a placebo-like condition in addition to no-contact controls or “treatment as usual” (see Boot et al., 2013, for a discussion on why even “active control” conditions can fail to generate an adequate placebo effect).
5. This study excluded from analysis 233 students in the experimental group who belong to arguably the most needy and vulnerable group among those living in poverty: those with brain injury, learning disability, neurodevelopmental disorder, ADHD or special education placement. *Individuals with these conditions in the control group were not excluded from analysis*. It is not possible to draw any comparison between study groups if people with known predictors of low performance were systematically removed from the intervention group while being allowed to remain in the control group. Socioeconomic status of the control group was also abstracted from school records rather than the family assessments given individually to students in the intervention group. Institutional Review Boards often dictate that vulnerable populations (e.g. those with severe learning disabilities) be excluded from studies to protect their safety or autonomy, but if they were enrolled in a study and given an intervention that purports to offer hope for the needy, then their data need to be analyzed as well. Perhaps more importantly, if we aim to draw conclusions about how best to intervene on the cognitively vulnerable, then the developmentally disabled and delayed are clearly an important part of the picture.
6. The SMART protocol was designed to provide a standardized intervention that could be administered in a classroom, yielding benefits for as many recipients as possible without requiring individualized instruction. Although a generalizable intervention of this sort could certainly prove beneficial in terms of cost-effectiveness, the goal of generalizability should not obscure the need to adapt teaching and training strategies to the unique needs of each student. Some people may benefit from educational strategies that involve bolstering their own unique weaknesses, and others–particularly those with known cognitive deficits–may gain more from strategies that involve using their strengths to compensate for weaknesses. In light of this, many in the cognitive training field are exploring the use of adaptive interventions that are calibrated to participants' abilities and change over the course of the intervention. If the aforementioned populations with special needs are eventually included in studies of interventions like SMART, we anticipate that adaptability will be a necessary component for the success of the intervention.

Within the educational context, the contents of the SMART intervention appear to have high face validity, meaning that educational psychology experts are likely to agree that the skills targeted are those that students should be learning anyway. The issue of opportunity costs arises when we try to generalize beyond the classroom, however. Any time we decide to do something, we are closing off the possibility of doing something else; consensus indicates that time spent learning how to read deeply is more beneficial for cognitive development than time spent on social media or watching sitcoms on television. If concerned parents are asking themselves what their children should do after school, however, an open question remains: should students do their homework as previously instructed, or should they continue practicing materials from interventions like SMART? We already know that physical fitness is one of the most potent neuroprotective factors across the lifespan (Colcombe and Kramer, 2003; Hillman et al., 2008); if families are making decisions about how to spend their limited resources, we must be frank about not yet having evidence that spending lots of time and money on proprietary reasoning courses is any better for cognitive development than joining the after-school track team for free, where we know that they will benefit from the effects of aerobic exercise and social engagement. It could very well be the case that cognitive training interventions are beneficial to cognitive development. To make that claim confidently, however, more research is required. Further programmatic research on cognitive training interventions should also enable us to answer more specific questions concerning the “active ingredients” of any particularly efficacious intervention, the types of cognitive changes to expect after training, the necessary doses and any overtraining or side effects, and the populations for which cognitive training is likely to have a beneficial effect.

### Recommendations and conclusions

The discussion on cognitive training continues, as does the rigorous program of research about its benefits and limitations. The U.S. National Institutes of Health, National Science Foundation, and the research divisions of the Departments of Defense and Intelligence are all funding research to investigate the efficacy of cognitive training interventions and the brain mechanisms that support different aspects of adaptive, goal-directed behavior. Where should they go from here? As members of the cognitive training field who share both the optimism and skepticism of our colleagues, we offer what we believe are several key points to advance the field, many of which are already being implemented by labs like those at the Center for BrainHealth:

1. Acknowledge the ambiguity of null findings, and publicize prominent failures to reject the null hypothesis. Use Bayesian statistics (in addition to the Neyman-Pearson and Fisher frameworks) to evaluate the state of the evidence, especially when trying to demonstrate that groups are equal, or that there is no difference between two conditions.
2. Keep in mind that a failure to replicate a finding does not mean that the original study was fraudulent, or even spurious. It only means that if a “true” effect exists, the size of the effect and the influence of any confounding variables is not yet clear.
3. Carefully control for factors that can be easily manipulated by transient environmental conditions like motivation and expectancy. Ideally, a double-blind and randomized treatment assignment procedure would mitigate the risk of participants in the control group underperforming due to low expectancy, or the risk of researchers treating them differently than the group receiving the intervention of interest. If the control group can not be led to believe they have a similar chance of improving to that of the intervention group, then their expectations of change should be measured.
4. In designing studies and randomizing participants, include (and statistically model) variability in stable confounding factors like developmental delay, traumatic brain injury and ADHD.
5. Use assessment batteries that include diverse tests of cognitive performance, such that the possibility of changes (and even decreases) in performance after training can be measured in cognitive domains other than the ones in which the experimenters expect improvement.

The most striking finding reported by Gamino and colleagues is that students are able to learn despite the environmental obstacles to success that confront them. That is reason enough to have hope. How much they learn, from what baseline, and under what conditions to allow for optimum growth remains to be determined, and we are confident that the cognitive neuroscience and psychology community is well equipped to address these questions.

## Author contributions

JO contributed to the conception, design and drafting of this article. AB contributed to the conception, design and critical revision for important intellectual content.

### Conflict of interest statement

The authors declare that the research was conducted in the absence of any commercial or financial relationships that could be construed as a potential conflict of interest. The reviewer KS and the handling Editor declared their shared affiliation, and the handling Editor states that the process nevertheless met the standards of a fair and objective review
